# Correlation of reduced temporal muscle thickness and systemic muscle loss in newly diagnosed glioblastoma patients

**DOI:** 10.1007/s11060-022-04180-8

**Published:** 2022-11-17

**Authors:** Cecil ten Cate, Sandra M. H. Huijs, Anna C. H. Willemsen, Raphael C. O. S. Pasmans, Daniëlle B. P. Eekers, Catharina M. L. Zegers, Linda Ackermans, Jan Beckervordersandforth, Elisabeth P. M. van Raak, Monique H. M. E. Anten, Ann Hoeben, Alida A. Postma, Martinus P. G. Broen

**Affiliations:** 1 Master of Science in Medicine and Clinical Research, Maastricht University, Maastricht, The Netherlands; 2 Department of Neurology Zuyderland Medical Center, Heerlen, The Netherlands; 3 GROW-School for Oncology and Reproduction, Maastricht University, Maastricht, The Netherlands; 4 Department of Respiratory Medicine, NUTRIM School of Nutrition and Translational Research in Metabolism, Maastricht University Medical Center, Maastricht, The Netherlands; 5 Division of Medical Oncology, Department of Internal Medicine, Maastricht University Medical Center, Maastricht, The Netherlands; 6 Department of Radiation Oncology (Maastro), GROW School for Oncology and Reproduction, Maastricht University Medical Center, Maastricht, The Netherlands; 7 Department of Neurosurgery, Maastricht University Medical Center, Maastricht, The Netherlands; 8 Department of Pathology, Maastricht University Medical Center, Maastricht, The Netherlands; 9 Department of Neurology, Maastricht University Medical Center, P.O. Box 5800, 6202 AZ Maastricht, The Netherlands; 10 Department of Radiology, Maastricht University Medical Center, Maastricht, The Netherlands

**Keywords:** Temporal muscle thickness, Skeletal muscle area, Glioblastoma, Sarcopenia, Imaging marker

## Abstract

**Purpose:**

Reduced temporal muscle thickness (TMT) has recently been postulated as a prognostic imaging marker and an objective tool to assess patients frailty in glioblastoma. Our aim is to investigate the correlation of TMT and systemic muscle loss to confirm that TMT is an adequate surrogate marker of sarcopenia in newly diagnosed glioblastoma patients.

**Methods:**

TMT was assessed on preoperative MR-images and skeletal muscle area (SMA) was assessed at the third lumbar vertebra on preoperative abdominal CT-scans. Previous published TMT sex-specific cut-off values were used to classify patients as ‘patient at risk of sarcopenia’ or ‘patient with normal muscle status’. Correlation between TMT and SMA was assessed using Spearman’s rank correlation coefficient.

**Results:**

Sixteen percent of the 245 included patients were identified as at risk of sarcopenia. The mean SMA of glioblastoma patients at risk of sarcopenia (124.3 cm^2^, SD 30.8 cm^2^) was significantly lower than the mean SMA of patients with normal muscle status (146.3 cm^2^, SD 31.1 cm^2^, *P* < .001). We found a moderate association between TMT and SMA in the patients with normal muscle status (Spearman’s rho 0.521, *P <* .001), and a strong association in the patients at risk of sarcopenia (Spearman’s rho 0.678, *P* < .001).

**Conclusion:**

Our results confirm the use of TMT as a surrogate marker of total body skeletal muscle mass in glioblastoma, especially in frail patients at risk of sarcopenia. TMT can be used to identify patients with muscle loss early in the disease process, which enables the implementation of adequate intervention strategies.

**Supplementary Information:**

The online version contains supplementary material available at 10.1007/s11060-022-04180-8.

## Introduction

Glioblastoma is the most common malignant primary brain cancer in adults. Current treatment consists of maximal surgical resection followed by a combination of radiation- and chemotherapy, achieving a median survival of only 15 months [1]. Patients’ frailty is a key factor negatively influencing survival, alongside with older age, less extensive tumor resection, corticosteroid treatment at baseline and the absence of promotor methylation of the O^6^-methylguanine-DNA methyltransferase (MGMT) gene [2]. Current widely used instruments to assess patients’ frailty, in terms of clinical condition, are the Karnofsky Performance Status (KPS) and Eastern Cooperative Oncology Group (ECOG) Performance status. Although simple and useful, they are subject to bias, such as high interobserver variability[3].

The past 5 years, reduced temporal muscle thickness (TMT) has been studied as a potential prognostic imaging marker and objective tool to assess patients’ frailty in both recurrent as well as de novo glioblastoma patients. Some confirmed its prognostic value in newly diagnosed glioblastoma [4–9], whereas others rejected it [10–12]. In addition, some reported only a prognostic role in recurrent, but not newly diagnosed, glioblastoma patients [11, 13]. These varying results are probably due to the lack of established non-sex specific cutoff values, varying study sample size and high percentages of missing tumor data. Recently, in a hallmark study to overcome these problems and facilitate clinical implementation, Furtner et al. [8] classified glioblastoma patients as ‘at risk of sarcopenia’ or ‘with normal temporal muscle status’ based on sex-specific TMT cutoff values 2.5 standard deviation below a normative reference population, based on recommendations of the European Working Group on Sarcopenia in Older People (EWGSOP) [14, 15]. Patients at risk of sarcopenia had a significantly higher risk of a short time to progression (TTP) after finalizing first line multimodality treatment and death than patients with normal temporal muscle status [8]. Sarcopenia is a condition characterized by the loss of body skeletal muscle mass and function [16] and also a proven prognostic factor in several other, non-brain cancers [17–19]. Systemic muscle loss can be quantified by analysis of cross-sectional area of skeletal muscles at the level of the third lumbar vertebra (L3) on computed tomography (CT) scans, which currently is the preferred method in the majority of cancer studies [20]. Skeletal muscle area (SMA) at L3 is highly correlated with total body skeletal muscle mass and therefore used as a by proxy measurement of sarcopenia [21, 22]. Reduced TMT, however, reflects very specific local sarcopenia, and it is unknown if reduced TMT also reflects more widespread skeletal muscle loss throughout the body. Confirmation of such an association attributes to early detection of sarcopenia, enabling early development of preventative individualized exercise or nutritional strategies focused on muscle retention for glioblastoma patients at risk of sarcopenia.

Previous studies investigating TMT as a prognostic marker in glioblastoma patients postulate it as a surrogate marker for sarcopenia based on one single study. This study by Leitner et al. showed a high correlation between lumbar SMA and TMT [23]. Their study was conducted in a population of lung cancer and melanoma patients with brain metastasis in an advanced disease setting. Our purpose was to validate these findings, by investigating the correlation between reduced TMT and systemic muscle loss, defined as low lumbar SMA, in newly diagnosed glioblastoma patients. Doing so, we aim to confirm that TMT is an adequate surrogate marker for total body skeletal muscle mass in this specific population.

## Methods

### Patient selection

A Dutch multicentre retrospective study was performed at the Maastricht University Medical Center+ (MUMC+) and Zuyderland Medical Center (ZMC). From an existing genotyped glioma database covering routine clinical diagnostics, data from newly diagnosed glioblastoma patients diagnosed or treated in MUMC + or ZMC between 2006 and 2020 were retrieved. Patients with glioblastoma (WHO grade 4), isocitrate dehydrogenase (IDH) wildtype, both MGMT hypermethylated or unmethylated were included. Patients with adequate preoperative brain Magnetic Resonance (MR) imaging as well as preoperative diagnostic abdominal CT scans were selected for this study. Patient characteristics and clinical data were collected from medical records as previously described [9].

### Assessment of temporal muscle thickness

TMT measurements were performed on axial isotropic (1 × 1 × 1 mm) contrast-enhanced T1-weighted MR images, which were routinely performed for neurosurgery navigation on the same day or one day before surgery, as previously described [9]. In short: the MR plane was oriented parallel to the anterior commissure-posterior commissure line. TMT was measured in mm perpendicular to the long axis of the temporal muscle at the level of the Sylvian fissure (anterior-posterior hallmark) and the orbital roof (craniocaudal landmark). The thickness was assessed on the left and right side separately. Then, TMT measurements of each side were summed and divided by two, resulting in a mean TMT per patient. Based on the mean TMT sex-specific cut-off values from Furtner et al. [8], patients were classified as ‘patient at risk of sarcopenia’ (mean TMT ≤ 6.3 mm for men and ≤ 5.2 mm for women) or ‘patient with normal muscle status’ (mean TMT > 6.3 mm for men and > 5.2 mm for women).

### Assessment of skeletal muscle area at the lumbar level

**Fig. 1:**
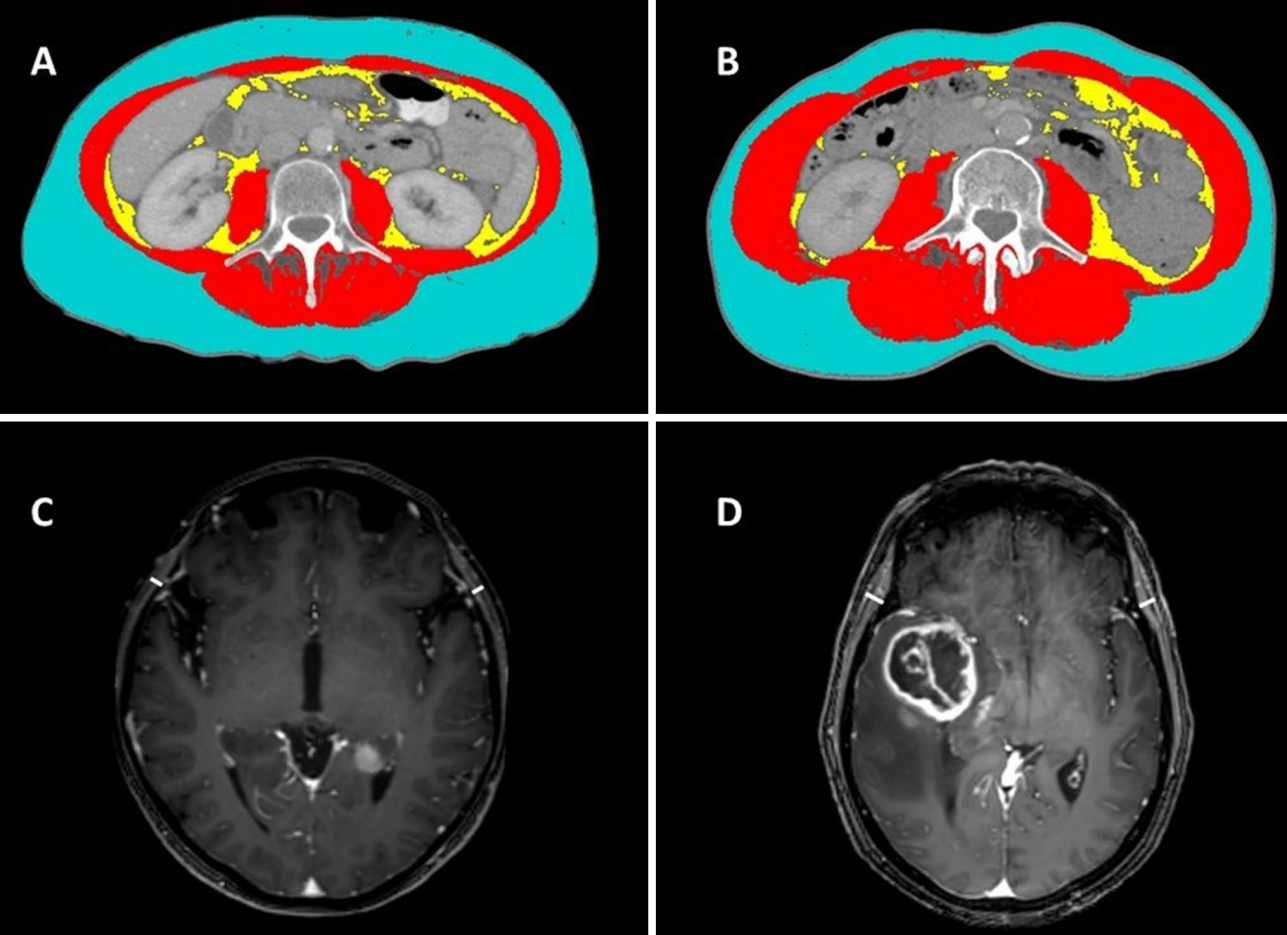
Examples of SMA assessment on transverse abdominal CT slices (**A**, **B**) and TMT assessment on axial T1-weighted contrast enhanced cranial MR images (**C**, **D**). **A** and **C** A 53 year old female at risk of sarcopenia according to TMT assessment (SMA = 76 cm^2^; TMT = 3.4 mm). **B** and **D** A 63 year old female with normal muscle status according to TMT assessment (SMA = 140 cm^2^; TMT = 8.3 mm). Red: skeletal muscle; Yellow: visceral fat; Blue: subcutaneous fat

An estimation of body skeletal muscle mass composition was based on measurement of skeletal muscle area (SMA) in cm^2^ at L3 on abdominal CT scans. For every patient, a single slice was manually selected from the pre-operative venous-phase CT scan on the level of L3 where both transverse processes were depicted. CT scans were assessed by one reviewer (CC) using Slice-O-Matic software v5.0 (Tomovision, Montreal, Canada) trained by a researcher with known prior experience with both the software as well as the SMA measurement (ACHW) [24]. Pre-established thresholds of Hounsfield units between − 29 and 150 for skeletal muscle were used. The reviewer was blinded to patient characteristics and mean TMT values at the time of SMA assessment. Examples of TMT assessment on brain MR images and SMA assessment on CT scans at the level of the third lumbar vertebra are provided in Fig. 1.

### Statistical analysis

Descriptive statistics were calculated for all patients. The statistical significance of differences between groups in mean TMT and in mean SMA was assessed using the Mann-Whitney U test. Correlation between mean TMT and SMA was assessed using Spearman’s rank correlation coefficient. A correlation coefficient of (−)0.8 to (−)1 was interpreted as a very strong association, of (−)0.6 to (−)0.8 as strong, of (−)0.4 to (−)0.6 as moderate, and of (−)0.2 to (−)0.4 as a low association. There was no association for a correlation coefficient of 0 to (−)0.2. Statistical analysis was performed using IBM Statistical Package for the Social Sciences, version 25 (SPSS, Chicago, Illinois). A two-tailed *P*-value of < 0.05 was considered statistically significant. In addition, SMA measurements of the total study population were compared with published data on a normative reference population [25].

## Results

### Cohort characteristics

**Fig. 2:**
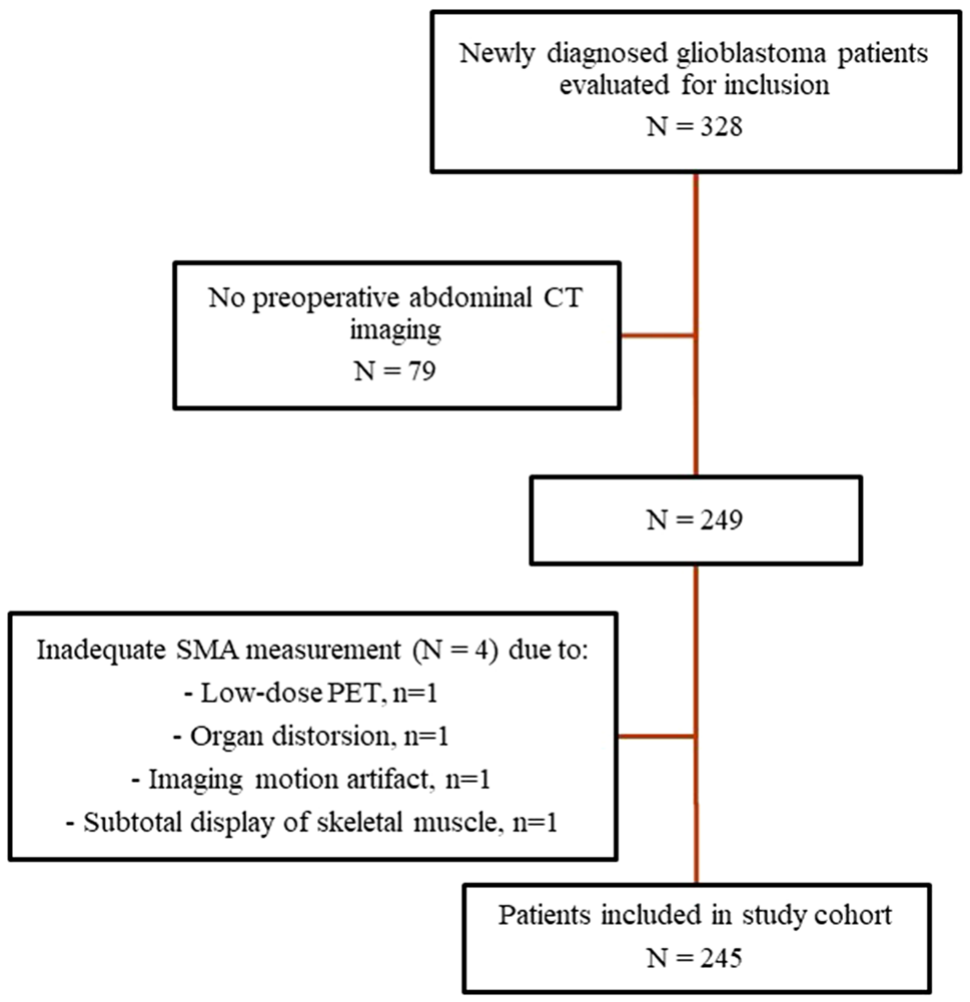
Flowchart of the patient selection process

The final study cohort consisted of 245 patients (Fig. 2). Their characteristics are listed in Table 1. Sex-specific mean TMT cutoff values were used to separate the cohort into patients at risk of sarcopenia (n = 39, 15.9%) and patients with normal muscle status (n = 206, 84.1%) [8].

**Table 1:** Patient characteristics

Variables	Study cohort (n = 245)
**Gender**	
Male, n (%)	156 (63.7)
Female, n (%)	89 (36.3)
**Mean age at diagnosis, years (SD**)	64.6 (9.8)
**MGMT hypermethylation**	
Yes, n (%)	96 (36.3)
No, n (%)	149 (63.7)
At risk of sarcopenia*	
Yes, n (%)	39 (15.9)
No, n (%)	206 (84.1)

Sarcopenia at risk is defined as a TMT cutoff value of ≤ 6.3 mm for men and ≤ 5.2 mm for women, which is 2.5 SD below the mean TMT value of a normative reference population [8].

*N* number; *SD* standard deviation, *MGMT* O6-methylguanine-DNA methyltransferase

### Temporal muscle thickness

All patients had measurements for both right and left TMT. The mean TMT of all patients was 7.7 mm (SD 1.9 mm), with a mean TMT in the subgroups of patients at risk of sarcopenia and patients with normal muscle status of 5.0 mm (SD 0.9 mm) and 8.2 mm (SD 1.5 mm), respectively (P < .001). Male patients had a mean TMT of 8.3 mm (SD 1.7 mm) which was significantly higher than the mean TMT of 6.6 mm (SD 1.6 mm) in female patients (*P* < .001).

### Skeletal muscle area at the lumbar level

**Fig. 3:**
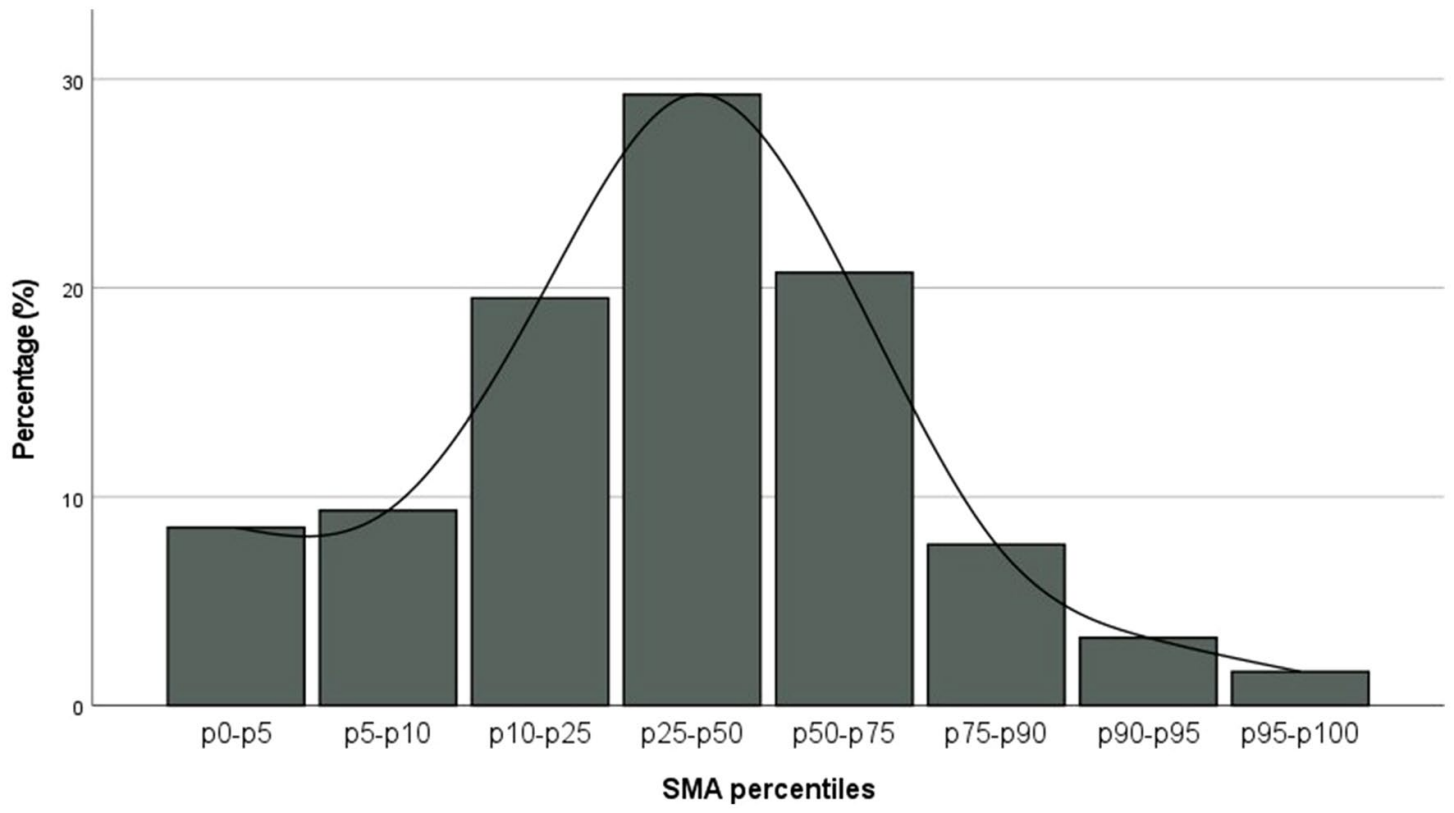
Bar chart of the SMA distribution in percentiles of the study population according to the sex specific percentiles for SMA drawn up by van der Werf et al. [25] based on a healthy population

The mean SMA of all patients was 142.8 cm^2^ (SD 32.0 cm^2^). Female patients had a mean SMA of 110.7 cm^2^ (SD 18.5 cm^2^), which was significantly lower than the mean SMA of 161.1 cm^2^ (SD 22.1 cm^2^) in male patients (*P* < .001). The mean SMA of glioblastoma patients at risk of sarcopenia (124.3 cm^2^, SD 30.8 cm^2^) was significantly lower than the mean SMA of patients with normal muscle status (146.3 cm^2^, SD 31.1 cm^2^, *P* < .001). We compared our SMA findings with reference values from a healthy population by using gender specific percentiles for skeletal muscle parameters [25]. The bar chart of the SMA distribution of our study population is compared with the reference population displayed as baseline normal distribution. Our studied population visually shows a relatively greater number of small SMA values at the lower percentiles compared to the normal reference population (Fig. 3).

### Correlation TMT and abdominal muscle area

A scatterplot of the correlation between the mean TMT and SMA is shown in Supplementary Fig. 1. The Spearman correlation coefficient between mean TMT and SMA in the total study population is 0.537 *(P =* .000), representing a moderate correlation. By dividing the population in subgroups with ‘normal muscle status’ and ‘at risk of sarcopenia’ based on mean TMT cutoff values, we found also a moderate association in the normal muscle group (0.521, *P <* .001) but a highly significant strong correlation between SMA and TMT measurements in patients at risk of sarcopenia (0.678, *P* < .001).

## Discussion

Our results confirm the use of TMT as a surrogate marker of total body skeletal muscle mass in glioblastoma patients at risk of sarcopenia. TMT can be used to identify patients with muscle loss early in the disease process, attributing to early detection of sarcopenia, enabling early development of preventative individualized intervention strategies for glioblastoma patients.

Only a few studies have investigated the correlation between TMT and SMA [23, 26, 27]. One study found a moderate correlation (r = .57) between TMT and psoas muscle area in trauma patients [26]. Although the correlation strength is comparable to our overall study population, there are some important differences. For example, Ranganathan et al. used CT imaging instead of standardized MR imaging to assess TMT values in a younger, non-cancer population[26]. Intriguingly, they found a stronger correlation in a more ‘frail’ subgroup, which is in line with our findings of a stronger correlation in glioblastoma patients in the ‘at risk of sarcopenia’ group, compared to the ‘normal muscle group’ based on TMT. This might indicate that especially in patients with lower muscle mass, the use of TMT as a surrogate marker of sarcopenia is most valid. Another study [27], found a strong correlation between TMT and calf circumference (0.608) and a moderate correlation with arm muscle circumference (0.433). However, TMT was assessed by ultrasound and only elderly (mean age 81 years) without malignancy were assessed, which is not representative for a population of glioblastoma patients. Only one study investigated the correlation between TMT and SMA in cancer patients [23]. Leitner et al. [23] analyzed the SMA at the level of the third lumbar vertebra and correlated these values with TMT on MR images of the brain in two cohorts of lung cancer (n = 93) and melanoma (n = 61) patients with brain metastases. They found a strong correlation between mean TMT and SMA (0.733) and concluded that TMT is a useful surrogate parameter for the estimation of skeletal muscle mass in patients with brain metastases. The strong correlation is comparable to the effect found in our subgroup of glioblastoma patients at risk of sarcopenia, but higher than the moderate correlation found in the normal muscle group. A possible explanation could lie in the fact that the study population of Leitner et al. already had an advanced stage of cancer at time of measurement, probably affected by several previous lines of treatment. In contrast, we assessed TMT and SMA at time of diagnosis, prior to surgery or any treatment. It is well known that cancer treatments and advanced disease stage contribute to muscle wasting. This is reflected by the lower mean SMA compared to our population (133 cm^2^ vs 143 cm^2^). As mentioned before, correlation between TMT and SMA seems to be stronger in frail or patients at risk of sarcopenia, which probably substantiates the high correlation in the study of Leitner et al. [23].

We compared patients’ SMA at time of diagnosis with a representative healthy population. We found that already at diagnosis more patients than expected have lower SMA values (Fig. 3). Because of our retrospective study design, we could not unravel if muscle loss at baseline is a consequence of the disease symptoms (e.g. impaired mobility due to paresis) or contrary a risk factor for the development of a glioblastoma (e.g. frail patients are more prone to develop an inflammatory process facilitating gliomagenesis [28, 29]). In addition, it can also not be ruled out that the glioblastoma micro-environment plays a role in inducing systemic inflammation facilitating sarcopenia. ​Although glioblastoma is historically not considered a ‘systemic’ disease, evidence of glioblastoma-derived mechanisms of, for example, systemic immunosuppression is emerging in the past decade [30]. Interestingly, skeletal muscle is postulated as potential central link between sarcopenia and immune senescence in elderly [31], which further supports the role of skeletal muscle loss in immune regulatory processes.

Whatever the cause is, glioblastoma patients at risk of sarcopenia have a higher risk of a short TTP after finalizing first line multimodality treatment and death than patients with normal muscle status, and also a higher risk of early discontinuation of treatment [9]. Glioblastoma patients might benefit from targeting muscle loss early in the diagnostic or treatment process. Interventions such as individualized exercise programs or nutritional advises such as a protein-rich diet [32], could reverse or stall the process of muscle wasting and improve treatment completion and survival. In addition, TMT might be used as an objective parameter of patients’ frailty, which can help physicians in dosing treatment or considering patient inclusion in clinical trials. Prospective studies are warranted to validate our findings and facilitate implementation in daily practice.

Our study has limitations. First, SMA was assessed only once by a single observer. To increase concordance CC was trained by a researcher (ACHW) with proven experience in SMA determination. The first 30 SMA assessments were directly observed by the trainer and the remaining assessments were checked at random [24]. In addition, we relied on previously published data which showed an excellent intra- and inter-observer agreement in determining SMA on abdominal CT scans [33]. Second, we used only a single abdominal slice (L3) as a surrogate marker of total skeletal muscle mass. Although it is postulated that the L3 level is the best by proxy measurement of skeletal muscle [34, 35], it doesn’t account for body composition differences caused by, for example ethnicity. However, we believe that the current method provides a good estimation of skeletal muscle mass with minimal effort and considerable accuracy. Developing technologies, especially deep learning and machine learning methods, will probably increase accuracy of muscle volume estimations in the nearby feature. A third limitation is the retrospective design, which made it not possible to investigate muscular strength or biochemical markers suck as systemic inflammation known to affect muscle mass. Additional longitudinal prospective studies are needed to confirm the correlation between radiologic features with muscle function, which is a key feature of true sarcopenia. Additionally, longitudinal studies will confirm if patients labeled at risk of sarcopenia eventually develop sarcopenia throughout the disease and treatment course. Fourth, 245 out of 328 patients had a preoperative abdominal CT scan. Imaging of the abdomen is not incorporated in international guidelines as a mandatory part of the diagnostic process of a presumed primary brain tumor. However, at MUMC + and ZMC it is common practice to perform abdominal and thoracic CT imaging to rule out other causes, such as a brain metastasis of a non-CNS- primary tumor, e.g. lung or breast cancer. In case of clear radiologic features of a glioblastoma, e.g. a butterfly glioma, no additional CT imaging is performed, which explains why not all patients underwent CT imaging preoperatively.

## Conclusion

Our study confirms the use of TMT in newly diagnosed adult glioblastoma patients as an adequate surrogate marker of total body skeletal muscle mass. This correlation is especially strong for frail patients at risk of sarcopenia. Although further prospective studies are needed, identifying these patients early in the disease process might open up opportunities to implement exercise or nutritional strategies to reverse or stall the process of muscle loss. Ultimately, this hopefully improves the percentage of patients completing treatment or receiving a second line treatment with improvement of survival of this devastating disease.

### Supplementary Information

Below is the link to the electronic supplementary material.

Supplementary material 1 (DOCX 121 kb)
