# Supplementary material for: Correlation of reduced temporal muscle thickness and systemic muscle loss in newly diagnosed glioblastoma patients
Source: J Neurooncol. 2022 Nov 17;160(3):611–8. doi: 10.1007/s11060-022-04180-8 (PMC9758090; doi:10.1007/s11060-022-04180-8)
Supplement: Supplementary file 1 — Supplementary material 1 (DOCX 121 kb) [file 11060_2022_4180_MOESM1_ESM.docx]

**Supplementary information**


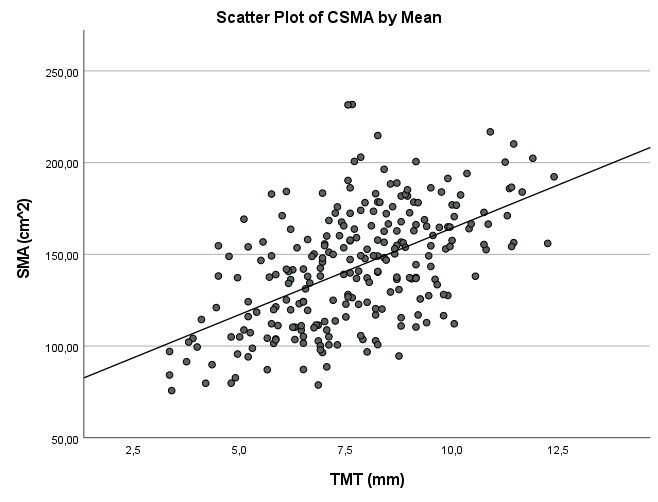


**Supplementary figure 1.** Scatterplot of the correlation between temporal muscle thickness (TMT) and skeletal muscle area (SMA).


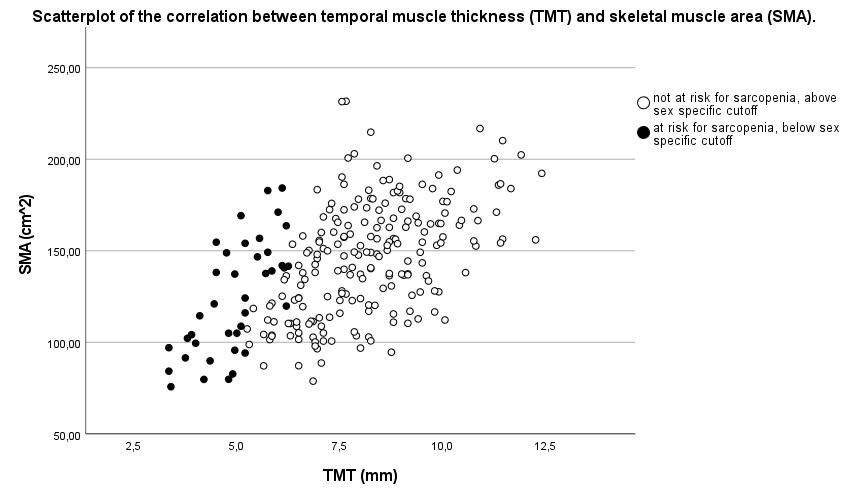


**Supplementary figure 2.** Scatterplot of the correlation between TMT and SMA. ‘At risk for sarcopenia’ (black) and ‘not at risk for sarcopenia’ (white) according to TMT sex-specific cut-off values.
